# Evolutionary consequences of shifts to bird-pollination in the Australian pea-flowered legumes (Mirbelieae and Bossiaeeae)

**DOI:** 10.1186/1471-2148-14-43

**Published:** 2014-03-07

**Authors:** Alicia Toon, Lyn G Cook, Michael D Crisp

**Affiliations:** 1 The University of Queensland, School of Biological Sciences, Brisbane Qld 4072, Australia; 2 Research School of Biology, The Australian National University, Canberra ACT 0200, Australia

**Keywords:** Pollination syndrome, Adaptive radiation, Ancestral state reconstruction, Diversification

## Abstract

**Background:**

Interactions with pollinators are proposed to be one of the major drivers of diversity in angiosperms. Specialised interactions with pollinators can lead to specialised floral traits, which collectively are known as a pollination syndrome. While it is thought that specialisation to a pollinator can lead to either an increase in diversity or in some cases a dead end, it is not well understood how transitions among specialised pollinators contribute to changes in diversity. Here, we use evolutionary trait reconstruction of bee-pollination and bird-pollination syndromes in Australian egg-and-bacon peas (Mirbelieae and Bossiaeeae) to test whether transitions between pollination syndromes is correlated with changes in species diversity. We also test for directionality in transitions that might be caused by selection by pollinators or by an evolutionary ratchet in which reversals to the original pollination syndrome are not possible.

**Results:**

Trait reconstructions of Australian egg-and-bacon peas suggest that bee-pollination syndrome is the ancestral form and that there has been replicated evolution of bird-pollination syndromes. Reconstructions indicate potential reversals from bird- to bee-pollination syndromes but this is not consistent with morphology. Species diversity of bird-pollination syndrome clades is lower than that of their bee-pollination syndrome sisters.

We estimated the earliest transitions from bee- to bird-pollination syndrome occurred between 30.8 Ma and 10.4 Ma. Geographical structuring of pollination syndromes was found; there were fewer bird-pollination species in the Australian southeast temperate region compared to other regions of Australia.

**Conclusions:**

A consistent decrease in diversification rate coincident with switches to bird pollination might be explained if greater dispersal by bird pollinators results in higher levels of connectivity among populations and reduced chances of allopatric speciation.

The earliest transitions overlap with the early diversification of Australian honeyeaters – the major lineage of pollinating birds in Australia. Our findings are consistent with the idea that environment and availability of pollinators are important in the evolution of pollination syndromes. Changes in flower traits as a result of transitions to bird-pollination syndrome might also limit reversals to a bee-pollination syndrome.

## Background

The extraordinary radiation of flowering plants (angiosperms) accounts for about 91% of all plant species and forms the foundations of terrestrial biodiversity [1,2]. This expansive evolution of species and morphological diversity has been attributed, at least in part, to interactions with animals [3,4] – particularly changes in specialist pollinators that might drive diversification [5-7]. There is good evidence that shifts between specialized pollinators are correlated with increased diversification in some angiosperm groups and decreases in others [8,9].

**Figure 1:**
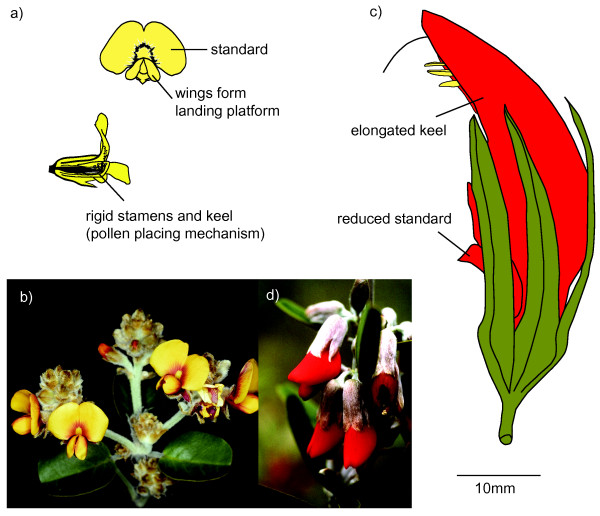
**Pollination syndromes.** Bee-pollination syndrome: **a)***Dillwynia uncinata*, showing the wing and keel that together act as a trigger mechanism. When a bee pushes down on the wings of a pea flower, the wings separate and trigger the sexual parts to rise out of the keel and deposit pollen from the anthers onto the bee. **b)** Flowers of *Gastrolobium pyramidale*. Bird-pollination syndrome: **c)***Leptosema aphyllum*, showing the resupinate orientation and large size. **d)** Flowers of *Gastrolobium rubrum*. Figure a) adapted from Gross [16].

The vast majority of flowering plants are pollinated by animals [10], including insects, birds, bats and other vertebrates, and there is good evidence that preferences of pollinators for different flower forms can lead to reproductive isolation in plants [11]. When interactions are specialized, the plant typically exhibits a pollination syndrome – a suite of floral characteristics that attract and provide food resources for a particular pollinator or pollinator guild [12,13]. For example, a bird-pollination syndrome typically involves large, red, tubular flowers, copious nectar and sexual parts positioned to deposit pollen on the bird (Figure 1). In contrast, bee-pollination syndromes have predominantly yellow, white or blue flowers (most hymenopteran pollinators have difficulty in discerning red flowers with a reflectance spectrum above 585 nm because they will not stand out from the green foliage [14,15]), a landing platform and guide marks and nutrient-rich nectar (Figure 1).

**Figure 2:**
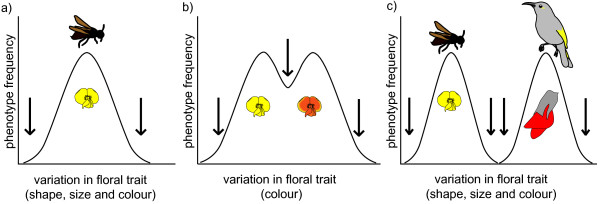
**Selection on flower colour and shape exerted by pollinators.** Example of how discrimination on floral traits by pollinators can limit divergence away from a pollination syndrome through stabilising selection or promote divergence towards a new pollination syndrome. Distributions represent the frequency of phenotypic variation within syndromes and arrows show phenotypes selected against by pollinators. Bee pollinators select for **a)** yellow flower colour, open shape and small size, limiting divergence away from the bee-pollination syndrome. **b)** An increase in pollination by birds in part of the species range might promote divergence in floral traits by selecting for flowers with a greater proportion of red (preferred by birds and less attractive to bees), followed by **c)** selection for larger size and pendular orientation.

It has been shown that pollination syndromes are integrated and are subject to selection that limits divergence in particular component traits away from some optimum (stabilizing selection, e.g., Figure 2) [17]. Nevertheless, there appear to have been many transitions between specialised pollination syndromes during the diversification of the angiosperms [18-20] but it appears that not all transitions are equally likely. Directional bias in transitions between specialised pollination syndromes, e.g., from bee-pollination to bird-pollination, has been indicated in several studies [18-20]. Such a bias could be caused by a structural-functional constraint preventing reversal of the complex trait of bird-pollination syndrome to a bee-pollination syndrome (an evolutionary ‘ratchet mechanism’ that facilitates transitions in one direction, [18,19]). For example, a flower specialised for bird pollination could be so large, with sexual parts so well separated, that a small insect (such as a small bee) could not effectively transfer pollen and thus would not exert disruptive selection on floral traits [19]. Similarly, it has been suggested that nectar in *Aquilegia* with floral spurs adapted to long-tongued pollinators (hummingbirds or hawkmoths) is inaccessible to short-tongued bees, which therefore do not exert selection on spur length [18]. A directional bias could also reflect a historical change in abundance of pollinator guilds, e.g., nectar-feeding birds might have evolved later than pollinating bees, or geographical variation in the distribution of pollinators caused by environmental heterogeneity, e.g., climatic factors that vary with altitude [21].

The legume tribes Mirbelieae and Bossiaeeae (the Australian “egg-and-bacon” peas) are a good system for addressing questions about floral transitions. Together, they comprise a diverse endemic Australian lineage in which species are specialised for either bee or bird pollination (Figure 1), as indicated both by direct observation [22-26] and by morphological syndromes [27,28]. Here we use comparative analyses of dated molecular phylogenies to address two questions:

1. Is pollination syndrome correlated with species diversity? If pollinators are influencing angiosperm diversification, there might be observable differences in diversity after shifts in pollinator.

2. Are all shifts equally likely? It would be expected that, if pollinator use is correlated with environment or if there is an evolutionary ratchet mechanism, there would be asymmetry in the directions of pollinator shifts.

## Methods

We sampled about half of the 700 known species of Mirbelieae and Bossiaeeae (Fabaceae, Australian egg-and-bacon peas), which are comprised of major endemic Australian pea genera such as *Daviesia*, *Bossiaea*, *Pultenaea*, *Mirbelia* and *Gastrolobium*. Trees were rooted with outgroups that included the likely sister group (*Hypocalyptus*), and other taxa sampled from across the legumes using Wojciechowski *et al*. (2004) [29] as a guide to relationships. Sequences of two cpDNA loci (*ndhF* and *trnL-trnF*) and one nrDNA locus (ITS1, 5.8S and ITS2) were obtained from our previous studies ([30] and references cited therein). DNA sequences were edited using Sequencher v4.5 (GeneCodes) and aligned manually in Se-Al v2.0a11 [31]. Parts of the ITS and *trnL-trnF* sequences that were not confidently aligned across the more distantly related terminals were offset or omitted. Ambiguity in aligning *trnL-trnF* and ITS prevented the use of outgroups more distantly related than *Baphia*.

### Phylogenetic analysis

Phylogenies were estimated for each locus using maximum likelihood (ML) in GARLI v0.951 [32] and a Bayesian MCMC search using MrBayes v3.1.2 [33]. All phylogenetic analyses incorporated a GTR + I + G model. As the resulting topologies and branch lengths showed little difference between the two search methods, all subsequent analyses used the GARLI trees with the best likelihood scores. The *trnL-trnF* and *ndhF* data showed no supported differences in resolution and were combined into a single cpDNA partition for comparative analyses. Other specifics of analyses were as per Crisp and Cook [30].

Phylograms were transformed into chronograms, in which branch lengths were proportional to time, using penalized likelihood (PL) in r8s v1.71 [34] with smoothing parameters optimised by fossil-based cross validation. To estimate ages of nodes, several primary (fossil-based) and secondary calibration points were used, as described previously [30]. We used the dated phylogenies to infer the timing of the earliest transitions between pollination syndromes. Confidence intervals around stem nodes of bird-pollination syndrome clades (crown node ages represent the latest possible transition time) were estimated from the ITS data and from the combined cpDNA data using 100 ML bootstrap trees with the ‘profile’ function in r8s.

### Transitions between pollination syndromes

Ancestral states for the root and internal nodes were inferred from each phylogeny using parsimony-based models as implemented in Mesquite version 2.74 [35]: equal-weighted parsimony and Dollo parsimony (bird, once gained from bee, is irreversible). The “Dollo” model is based on the arguments that, once lost, a complex trait cannot revert to exactly the same form it was before the change [36,37]. Pollination syndrome may satisfy the criterion of a complex trait because flowers of different syndromes differ in multiple, integrative floral attributes, including colour, shape, nectar and orientation [12]. If evolution of floral morphology underlying a bird-pollination syndrome involves changes in multiple pathways, it might be expected that changes back to a bee-pollination syndrome might be difficult or, if it does occur, results in a morphology that is somewhat different from the ancestral bee-pollinated floral morphology.

### Differences in diversity

If shifts in pollination syndrome have contributed to the diversity of angiosperms, we might expect there to be observable differences in species diversity of clades exhibiting different pollination syndromes. Sister-clade comparisons are a good way of testing for species-richness differences if a sufficient number of pairs are available to provide power to the test. Sister clades share a single common ancestor and so any differences in species diversity must have arisen since their divergence from that ancestor. We tested whether there was a difference in species richness between clades with bird- and bee-pollination syndrome using a Wilcoxon signed-rank test of sister clades as implemented in GraphPad Prism 5.03, GraphPad Software, San Diego California USA, www.graphpad.com. Given that there were slight differences in topologies among trees derived from the different DNA regions, we conducted the sister-taxon comparisons separately using ML trees from both ITS and combined cpDNA. One sister pair of bird-bee-pollination syndrome was removed from the ITS analysis because of uncertain phylogenetic resolution. There was insufficient phylogenetic resolution to identify the sister to the bird-pollination syndrome clade (i.e., it was part of a polytomy) in *Gastrolobium* in both gene trees, so we combined the unresolved bee-pollination syndrome species into a single clade. Combining these clades did not bias the result because the total species diversity of the combined bee-pollination syndrome clades was less than that of the bird-pollination syndrome clade – our approach was conservative. To complement the sister-pair comparisons, we used the BiSSE approach [38] available in Mesquite v2.74 (with the Goldberg correction module, [39]) to test the null hypothesis that diversification rates remained constant through inferred transition events, i.e., that there was no difference in diversification rates of bee- and bird-pollination syndrome clades. We compared the models using Akaike information criterion (AIC) following Burnham [40], where the model with the lower score is a better fit.

### Geographic distribution of pollination syndromes

If different pollination syndromes are favoured under different ecological conditions, we might expect them to be represented differently in each major habitat. We tested whether there was geographic variation in the proportion of bird- and bee-pollination syndrome species richness as a proxy for an external ecological driver. We categorised all described species of Mirbelieae and Bossiaeeae as occurring in one or more of four biomes based on distribution data from Australia’s Virtual Herbarium (http://chah.gov.au/avh/index.jsp, accessed 11 Nov 2011) and regions described in [41,42]: southeast temperate, southwest temperate, central (arid), and tropical (monsoon tropics). We used a Chi-squared test to determine whether there was a difference in the proportion of bird-pollination syndrome species occurring in these regions.

## Results

**Figure 3:**
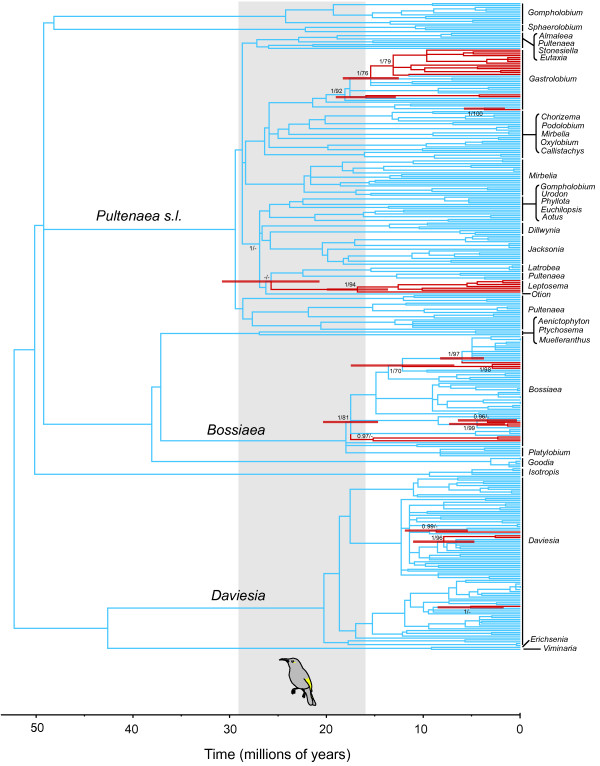
**Dating origins of bee- and bird-pollination syndromes using cpDNA.** Chronogram of Mirbelieae and Bossiaeeae derived from Maximum Likelihood analysis of combined cpDNA sequence data using PL rate smoothing. Parsimony trait reconstruction of bee- (blue) and bird- (red) pollination syndromes (no directional weighting) is shown on tree. Red bars show 95% confidence intervals of stem age of bird-pollination syndrome transitions and crown age of the earliest inferred bird-pollination syndrome transition (*Leptosema*). Posterior probabilities > 0.95 (*PP*) and bootstrap support > 70 (BS) are shown on stem and crown nodes of bird-pollination syndrome transitions. Where node support is lower than 0.95/70, *PP* and BS is also shown on the node above the transition. Inferred crown and stem age (95% highest probability density) of honeyeaters (Meliphagidae) is shaded grey.

**Figure 4:**
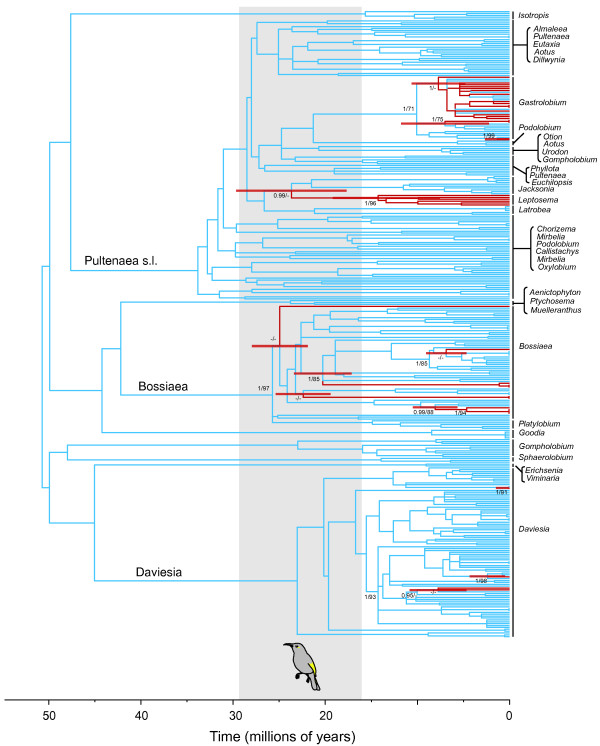
**Dating of origins of bee- and bird-pollination syndromes using ITS.** Chronogram of Mirbelieae and Bossiaeeae derived from Maximum Likelihood analysis of ITS sequence data using PL rate smoothing. Parsimony trait reconstruction of bee- (blue) and bird- (red) pollination syndromes (no directional weighting) is shown on tree. Red bars show 95% confidence intervals of stem age of bird-pollination syndrome transitions and crown age of the earliest inferred bird-pollination syndrome transition (*Leptosema*). Posterior probabilities > 0.95 (*PP*) and bootstrap support > 70 (BS) are shown on stem and crown nodes of bird-pollination syndrome transitions. Where node support is lower than 0.95/70, *PP* and BS is also shown on the node above the transition. Inferred crown and stem age (95% highest probability density) of honeyeaters (Meliphagidae) is shaded grey.

A bee-pollination syndrome is clearly reconstructed as ancestral in the Australian egg-and-bacon peas (Figures 3 and 4, Additional file 1: Figure S1 and Additional file 2: Figure S2). In all analyses, species with a bird-pollination syndrome are nested within clades of bee-pollinated taxa that are well supported by posterior probability (Bayesian) and bootstrap support (maximum likelihood) (Additional file 1: Figure S1 and Additional file 2: Figure S2). The earliest transition to bird-pollination syndrome was reconstructed on the stem leading to the *Leptosema* clade. We estimated the earliest transition occurred between 25.7 Ma (95% CI = 20.6-30.8 Ma; stem age) and 16.8 Ma (95% CI = 10.4-23.2 Ma; crown age) using cpDNA data and 23.6 Ma (95% CI = 16.8-29.6 Ma; stem age) and 13.4 Ma (95% CI = 7.6-19.2 Ma; crown age) using ITS data.

### Diversity

We compared diversity of sister clades differing in pollination syndrome and found that clades of species with bird-pollination syndrome had fewer species than their sister clade with bee-pollination syndrome (Wilcoxon signed-rank tests: combined cpDNA: n = 11, Z = -2.073, *P* = 0.038; ITS: n = 11, Z = -2.014, *P* = 0.044). The BiSSE model, which accounts for interaction between trait states and diversification rates, marginally supported the state-independent maximum likelihood (ML) models over state-dependent models (delta AIC = 3.96), although both models are still favoured where delta AIC < 4 [40].

### Shifts in pollination syndrome

**Figure 5:**
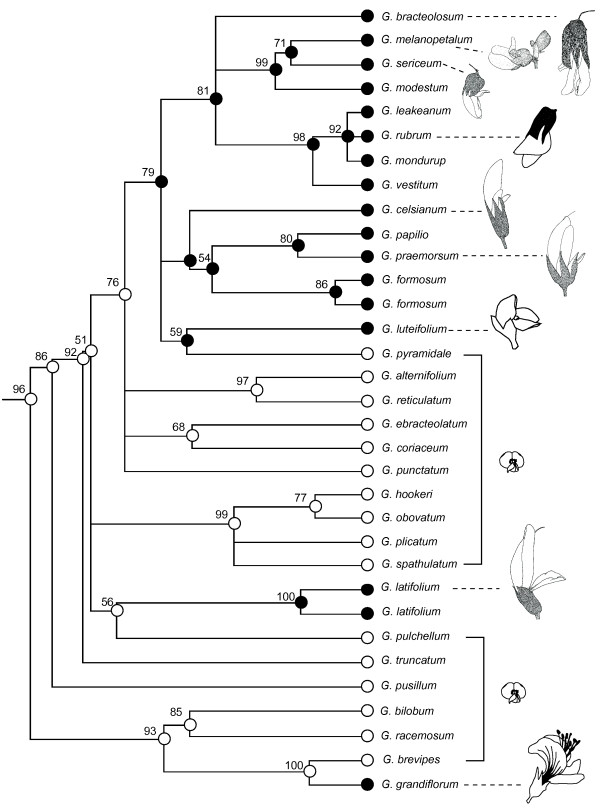
**Pollination syndrome in *Gastrolobium*.** Maximum likelihood tree of combined cpDNA sequences showing relationships among *Gastrolobium* species and inferred bee- and bird-pollination syndromes. Parsimony ancestral reconstruction of bee-pollination syndrome (white circles) and bird-pollination syndrome (black circles) is shown on the nodes. Bootstrap support is shown on the branches. Examples of flowers highlight the diversity in shape of the bird-pollination syndrome flowers.

Phylogenetic relationships in the egg-and-bacon peas differed between estimates using cpDNA and ITS, resulting in slightly different reconstructions of transitions between bee- and bird-pollination syndromes (Additional file 1: Figure S1 and Additional file 2: Figure S2). This uncertainty in reconstructing shifts of pollinator syndrome is centred around relationships within *Gastrolobium*, many of which do not have strong support (Additional file 1: Figure S1 and Additional file 2: Figure S2). The single inferred reversal in analyses of cpDNA involved a transition back to bee-pollination syndrome in *G. pyramidale* (Figure 5), but this node is not well supported and the alternative resolution, in which *G. pyramidale* is sister to a clade of bird-pollination syndrome species instead of within the clade, is not rejected. The five or six reversals inferred in ITS analyses are also within *Gastrolobium* and do not have strong support (Additional file 1: Figure S1 and Additional file 2: Figure S2). For example, *G. alternifolium* is placed with two bird-pollination syndrome species (*PP* = 0.96) but their position among other bird-pollinated species has low resolution and in the cpDNA analysis, G. *alternifolium* is placed with bee-pollination syndrome species (Additional file 1: Figure S1 and Additional file 2: Figure S2).

In all reconstructions, there were many more inferred shifts from bee- to bird-pollination syndrome than the reverse (Table 1). An assumption of no reversals from bird- to bee- pollination syndrome (Dollo’s law) resulted in one extra transition (14 vs 13) in analyses of cpDNA, and two extra transitions (20 vs 18) in analyses of ITS (Table 1).

**Table 1:** Number of transitions between bee- and bird-pollination syndromes inferred from maximum parsimony (MP) reconstruction methods using the ITS and cpDNA phylogeny

**Dataset**	**Model**	**Bee to bird**	**Bird to bee (reversals)**	**Total transitions**
ITS chronogram	MP (equal weighted) - MPR1	12	6	18
	MP (equal weighted) - MPR2	11	7	18
	Dollo parsimony	20	0	20
cpDNA chronogram	MP (equal weighted)	12	1	13
	Dollo parsimony	14	0	14

### Geographic distribution of pollination syndromes

There are proportionally fewer bird-pollination syndrome species of egg-and-bacon peas in the Australian southeast temperate region (SET) (about 1%) than any other region (Chi-squared = 15.108, df = 3, *P* = 0.0017), and no significant difference between proportions in the southwest temperate (5.9%), tropical (9.3%) and central (10.5%) regions (Chi-squared = 2.781, df = 2, *P* = 0.249).

## Discussion

Pea flowers of the subfamily of legumes classified as Faboideae (or Papilionoideae) arose about 58 Ma [43] and are thought to have evolved as a specialisation to pollination by bees [44]. Our reconstruction of a bee-pollination syndrome as ancestral for the Australian egg-and-bacon peas is consistent with it being the ancestral syndrome for the entire subfamily. The main bird pollinators of Australian egg-and-bacon peas, the honeyeaters (Meliphagidae), probably radiated between 15.9-29.4 Ma [45]. The earliest transitions to bird-pollination syndrome, which we estimated to have occurred *ca.* 10.4-30.8 Ma (cpDNA) and 7.6-29.6 Ma (ITS), mostly overlap with the radiation of honeyeaters. Thus, our results are consistent with this pollination syndrome being dependent on the availability of potential pollinators: bees of several families were present in Australia before the diversification of honeyeaters (e.g., [46,47]), and it appears that egg-and-bacon peas only switched to bird-pollination syndromes once the honeyeaters were present to exert selection pressure on flower morphology. The honeyeaters have a visual system that responds well to the red wavelengths [48], and multiple lineages of Australian plants, such as Proteaceae [49], appear to have developed floral syndromes that are coloured for the tetrachromic vision of honeyeaters after this group radiated [50].

Similarly to reports for some plants outside Australia (e.g., [51,52]), we found that lineages of the Australian egg-and-bacon peas exhibiting a bird-pollination syndrome have fewer species than their sister clades of bee-pollination syndrome species. Differences in species diversity are explained by changes in diversification rate, that is, in the rates of speciation and/or extinction. Pollination syndromes could directly influence either rate (speciation or extinction) by affecting genetic connectivity across a species’ range over time [53]. Different pollinators move pollen across different distances, thus affecting genetic connectivity across a species range (eg., [54,55]). Australian honeyeaters have been found to distribute pollen over distances of 10^3^-10^4^ metres whereas bees typically do so over 10^2^ metres [56-58], and bees transfer pollen between flowers less efficiently than birds [19]. Lower levels of pollen movement could lead to greater spatial genetic structure and increase the chance of speciation via allopatric speciation in bee-pollinated taxa compared to bird-pollinated taxa. The corollary of this is that bird-pollinated taxa should be subject to lower levels of differentiation in allopatry.

### Directional bias in transitions in pollination syndrome

We found multiple independent origins (replicated evolution) of bird-pollination syndromes within the Australian egg-and-bacon peas with few, if any, reversals back to a bee-pollination syndrome. This asymmetry in direction of pollinator transitions might have several underlying causes.

1. The floral morphology that suits pollination by birds might have resulted in sexual parts oriented in such a way that bees do not transfer pollen between flowers, and thus do not exert selection pressure on bird-pollination syndrome flowers. That is, the system represents an evolutionary ratchet mechanism where, once floral morphology has diverged sufficiently, reversions to the previous state are rare.

Although all reconstructions favoured at least one reversal from bird-pollination syndrome to bee-pollination syndrome, a Dollo model (no reversals) required only one or two additional switches in pollination syndrome (Table 1). Determining which reconstruction is most accurate cannot be determined from phylogenies alone (e.g., [59,60]) and additional information is required to test hypothesised pathways. Under a scenario of replicated evolution of a trait (here bird-pollination syndrome) it is expected that, in most cases, the trait will have evolved in slightly different ways because the lineages are independent, although they might be closely related. This is evident in parallel transitions to bird-pollination syndrome in egg-and-bacon peas. There are some commonalities, such as enlarged and/or red tubular corollas, but there are also lineage-specific differences, as expected. For example, *G. rubrum* and *G. bracteolosum* have a reduced standard, enlarged keel and are pendulous, whereas some others (*G. leakeanum*, *G. mondurup* and *G. vestitum*) have a flower shape more similar to a typical bee-pollination syndrome but are resupinate (turned upside down) and much larger (Figure 5). Others, such as *G. melanopetalum*, *G. sericeum* and *G. modestum,* have cream or black flowers that are inflated at the base, with a small keel and canaliculate (longitudinally grooved) standard.

Similarly, and as stated under Dollo’s model, if a complex trait is lost it is unlikely to re-evolve in exactly the same form. For example, some flowers that appear to have undergone reversals from zygomorphy (monosymmetry) to ancestral polysymmetry have been found to have vestiges of their zygomorphic past (e.g., asymmetrical stamen development in *Saintpaulia*) or a different form of polysymmetry (e.g., six petals instead of five) [61]. In all our analyses, *G. pyramidale* (bee-pollination syndrome) was reconstructed as having been derived from a lineage that exhibited bird-pollination syndromes. However, we do not think reversal is the best explanation. Firstly, the node was only weakly supported by either DNA region and it is possible that this taxon is sister to the bird-pollination syndrome clade rather than being derived from within it. Secondly, even if the topology is accurate, *G. pyramidale* has a floral morphology that is indistinguishable from other bee-pollination syndrome taxa in *Gastrolobium* – it looks the same as a typical bee-pollination syndrome member of the egg-and-bacon peas. Other species of *Gastrolobium* (e.g., *G. alternifolium*) that were inferred as a reversal of bird-pollination syndrome to bee-pollination syndrome also look the same as a typical bee-pollination syndrome. We think it is more likely that there have been more transitions to bird-pollination syndrome in this lineage, consistent with the Dollo model and the observation of multiple different forms of bird-pollination syndrome in the clade, and that *G. pyramidale* has not changed from its bee-pollination syndrome ancestry.

2. An alternative explanation to the bias in direction of pollination syndrome transitions might be that current environmental conditions favour bird pollination over bee pollination, and thus there is little selection favouring reversals. Orians and Milewski [62] postulated that Australia has more vertebrate-pollinated species than other continents because it has poor soils and ample sunshine. Under such conditions and with sufficient water, plants are able to produce excess carbohydrates and thus make copious quantities of nectar, which attracts large vertebrates [63]. The nectar produced on nutrient poor soil might be low in proteins [63], however, unlike insect-pollinators, vertebrates readily acquire proteins from other sources in their diet such as insects [62]. Our findings are partly consistent with this idea because we found greater proportions of bird-pollination syndrome egg-and-bacon peas in regions with poorest soils (southwest temperate, central and tropical Australia) compared with the more fertile southeast temperate region. However, the ability to make copious nectar cannot explain the transition to a bird-pollination syndrome, only that it is possible. Other ecological factors that favour bird over bee pollination must be present. For example, differences in abundance of pollinators across the landscape might drive the initial divergence needed for speciation [5,6], and classic studies on *Mimulus*[21] support this as a likely mechanism. Most Australian honeyeaters feed also on insects and are not limited to nectar [64], so they can persist in areas and through seasons when bees are absent. Pollinator abundance might also be influenced by the presence of other species of flowering plants. Coexistence of peas in a community with a high abundance of bird-pollinated taxa, such as in southwest Western Australia [25], might promote reproductive success in peas that switch to bird-pollination by increasing visitation of pollinators and providing shelter and nesting for birds, as suggested for banksias and eucalypts by He et al. [65].

## Conclusions

The Australian egg-and-bacon peas exhibit replicated evolution of bird-pollination syndromes, with little evidence that there have been switches back to an ancestral-type bee-pollination syndrome. Our reconstructions inferred that a shift in pollinator has repeatedly led to a decrease in diversification rate, with bird-pollination syndrome clades less species rich than their bee-pollination syndrome sisters. This might be explained if greater dispersal of bird-pollinators results in higher levels of connectivity and decreases the chance of allopatric speciation in bird-pollinated species. Further studies comparing gene flow and population structure will contribute towards an understanding of how different pollination specialisations have contributed to the diversification of angiosperms.

### Availability of supporting data

Additional file 1: Figure S1.

Additional file 2: Figure S2.

## Abbreviations

ML: Maximum likelihood; PL: Penalized likelihood; AIC: Akaike information criterion; PP: Posterior probabilities; BS: Bootstrap support; MP: Maximum parsimony; SET: Southeast temperate region; SWT: Southwest temperate region.

## Competing interests

The authors declare that they have no competing interests.

## Authors’ contributions

AT analysed the data and led preparation of the manuscript. LGC conceptualized the project and collected data. MDC conceptualized the project, collected and analysed data. All authors contributed to writing of the manuscript and read and approved the final manuscript.

## Supplementary Material

Additional file 1: Figure S1 Bayesian estimate of Mirbelieae and Bossiaeeae phylogeny using combined cpDNA sequence data showing clades that include both bird (red) and bee (black) pollinator syndromes. Full tree is shown on left with partial tree (bold in full tree) on right of a: *Gastrolobium*, b: *Bossiaea* and *Platylobium*, c: *Daviesia* and d: *Gompholobium, Urodon, Aotus, Euchilopsis, Phyllota, Dillwynia, Jacksonia, Leatrobea, Pultenaea* and *Leptosema*. Posterior probabilities (*PP* > 0.95) from Bayesian analysis and bootstrap support of bipartitions (BS > 0.70) from maximum likelihood analysis are shown on branches. Scale bar represents substitutions per site. Click here for file

Additional file 2: Figure S2 Bayesian estimate of Mirbelieae and Bossiaeeae phylogeny using ITS sequence data showing clades that include both bird (red) and bee (black) pollinator syndromes. Full tree is shown on left with partial tree (bold in full tree) on right of a: *Gastrolobium*, b: *Bossiaea* and *Platylobium*, c: *Daviesia* and d: *Jacksonia* and *Leptosema*. Posterior probabilities (*PP* > 0.95) from Bayesian analysis and bootstrap support of bipartitions (BS > 0.70) from maximum likelihood analysis are shown on branches. Scale bar represents substitutions per site. Click here for file
