# Supplementary material for: Evolutionary consequences of shifts to bird-pollination in the Australian pea-flowered legumes (Mirbelieae and Bossiaeeae)
Source: BMC Evol Biol. 2014 Mar 7;14:43. doi: 10.1186/1471-2148-14-43 (PMC4015313; doi:10.1186/1471-2148-14-43)
Supplement: Additional file 1: Figure S1 — Bayesian estimate of Mirbelieae and Bossiaeeae phylogeny using combined cpDNA sequence data showing clades that include both bird (red) and bee (black) pollinator syndromes. Full tree is shown on left with partial tree (bold in full tree) on right of a: Gastrolobium, b: Bossiaea and Platylobium, c: Daviesia and d: Gompholobium, Urodon, Aotus, Euchilopsis, Phyllota, Dillwynia, Jacksonia, Leatrobea, Pultenaea and Leptosema. Posterior probabilities (PP > 0.95) from Bayesian analysis and bootstrap support of bipartitions (BS > 0.70) from maximum likelihood analysis are shown on branches. Scale bar represents substitutions per site. [file 1471-2148-14-43-S1.pdf]

Additional file 1 Figure S1: Bayesian estimate of Mirbelieae and Bossiaeeae phylogeny using combined cpDNA sequence data showing clades that include both bird (red) and bee (black) pollinator syndromes. Full tree is shown on left with partial tree (bold in full tree) on right of a: *Gastrolobium*, b: *Bossiaea* and *Platylobium*, c: *Daviesia* and d: *Gompholobium, Urodon, Aotus, Euchilopsis, Phyllota, Dillwynia, Jacksonia, Leatrobea, Pultenaea* and *Leptosema*. Posterior probabilities (*PP* > 0.95) from Bayesian analysis and bootstrap support of bipartitions (BS > 0.70) from maximum likelihood analysis are shown on branches. Scale bar represents substitutions per site.

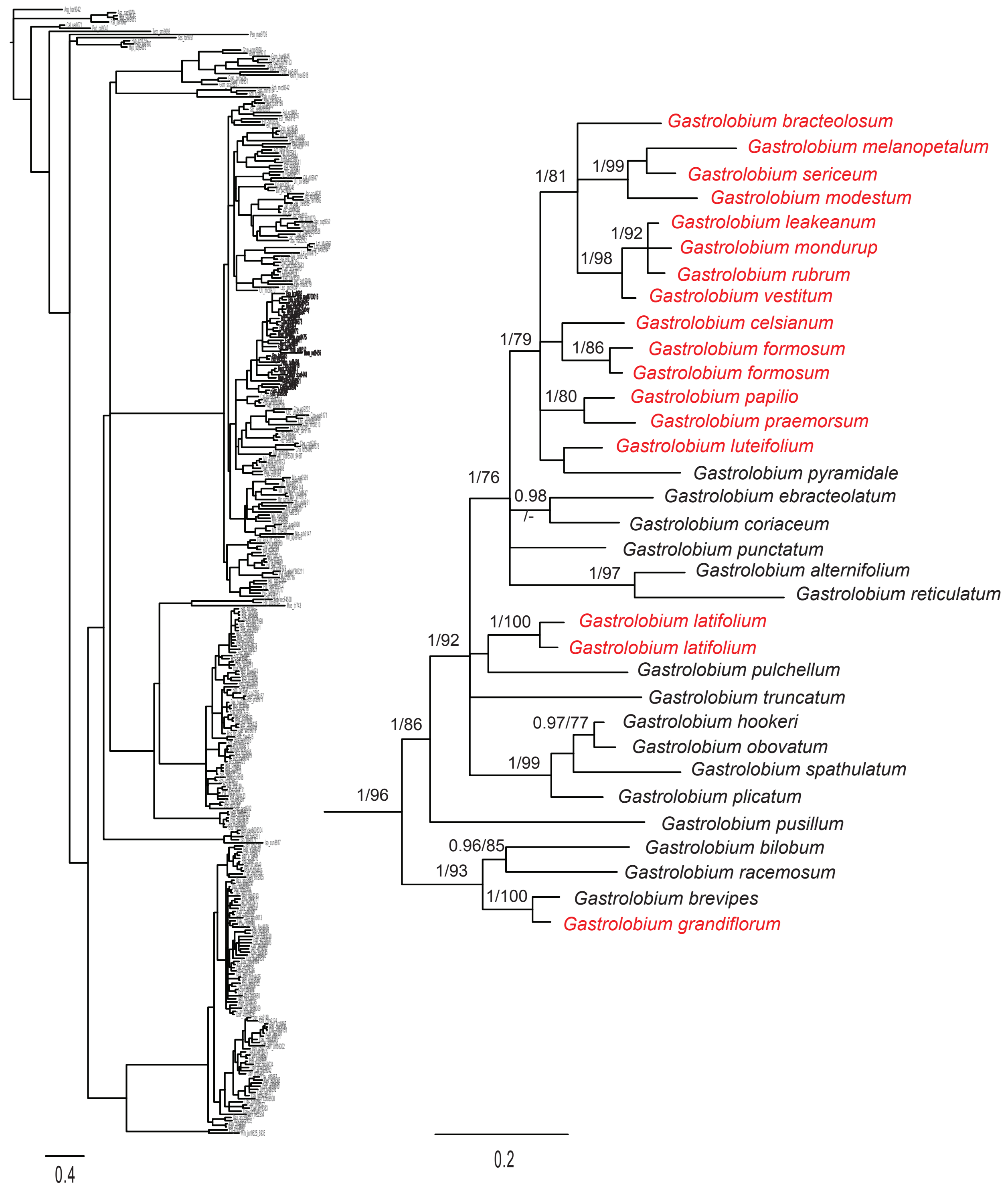
Gastrolobium bracteolosum
Gastrolobium melanopetalum
Gastrolobium sericeum
Gastrolobium modestum
Gastrolobium leakeanum
Gastrolobium mondurup
Gastrolobium rubrum
Gastrolobium vestitum
Gastrolobium celsianum
Gastrolobium formosum
Gastrolobium formosum
Gastrolobium papilio
Gastrolobium praemorsum
Gastrolobium luteifolium
Gastrolobium pyramidale
Gastrolobium ebracteolatum
Gastrolobium coriaceum
Gastrolobium punctatum
Gastrolobium alternifolium
Gastrolobium reticulatum
Gastrolobium latifolium
Gastrolobium latifolium
Gastrolobium pulchellum
Gastrolobium truncatum
Gastrolobium hookeri
Gastrolobium obovatum
Gastrolobium spathulatum
Gastrolobium plicatum
Gastrolobium pusillum
Gastrolobium bilobum
Gastrolobium racemosum
Gastrolobium brevipes
Gastrolobium grandiflorum
1/81
1/99
1/92
1/98
1/79
1/86
1/80
1/76
0.98
/-
1/97
1/100
1/92
1/86
0.97/77
1/99
1/96
0.96/85
1/93
1/100
0.4
0.2

Figure S1a

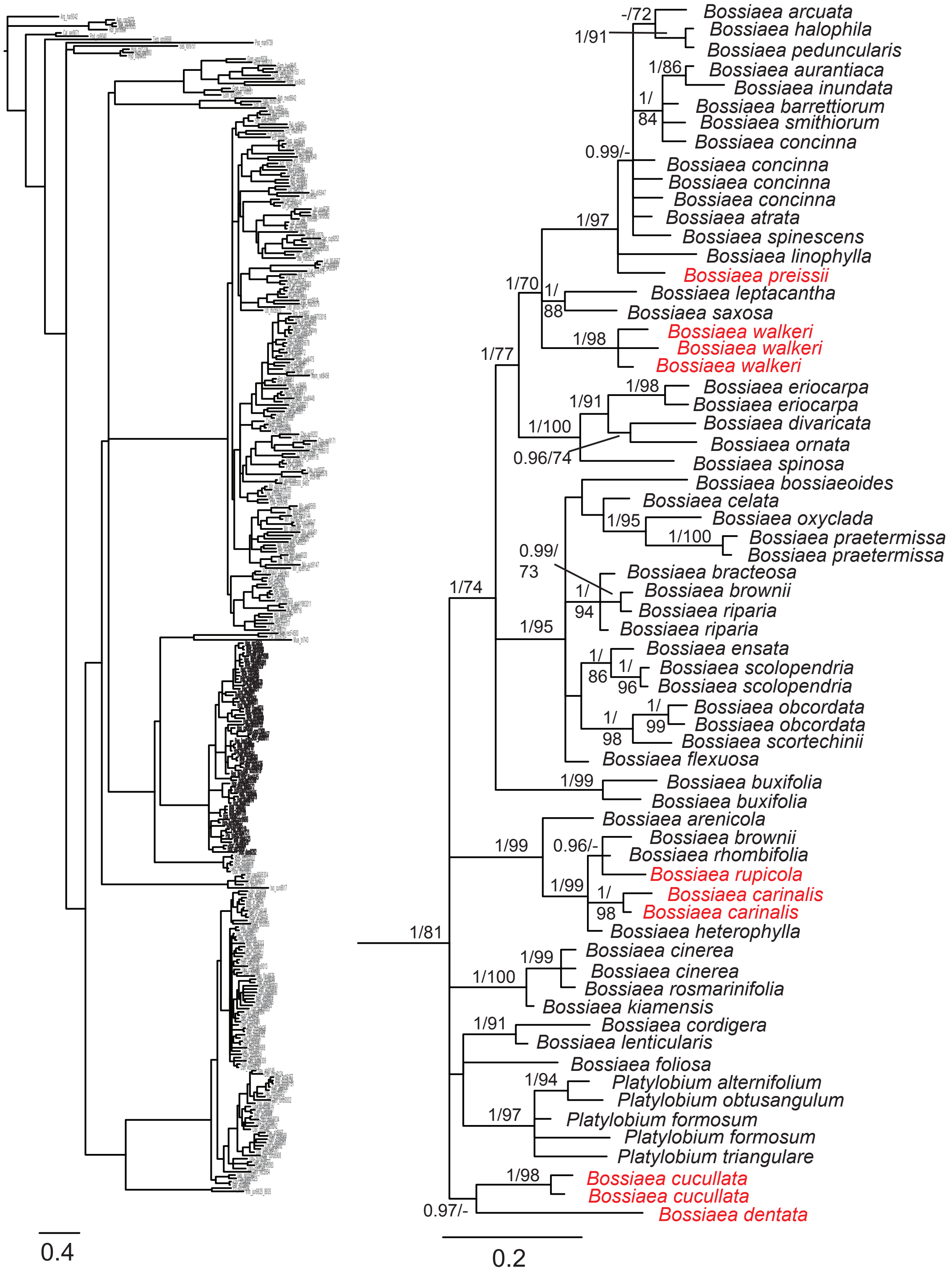

0.4
0.2
-/72
1/91
Bossiaea arcuata
Bossiaea halophila
Bossiaea peduncularis
1/86
Bossiaea aurantiaca
Bossiaea inundata
1/
84
Bossiaea barrettiorum
Bossiaea smithiorum
Bossiaea concinna
0.99/-
Bossiaea concinna
Bossiaea concinna
Bossiaea concinna
Bossiaea atrata
1/97
Bossiaea spinescens
Bossiaea linophylla
Bossiaea preissii
1/70
1/
88
Bossiaea leptacantha
Bossiaea saxosa
1/77
1/98
Bossiaea walkeri
Bossiaea walkeri
Bossiaea walkeri
1/98
Bossiaea eriocarpa
1/91
Bossiaea eriocarpa
Bossiaea divaricata
1/100
Bossiaea ornata
0.96/74
Bossiaea spinosa
Bossiaea bossiaeoides
Bossiaea celata
1/95
Bossiaea oxyclada
1/100
Bossiaea praetermissa
Bossiaea praetermissa
0.99/
73
Bossiaea bracteosa
1/
94
Bossiaea brownii
Bossiaea riparia
1/74
Bossiaea riparia
1/95
Bossiaea ensata
1/
86
1/
96
Bossiaea scolopendria
Bossiaea scolopendria
1/
99
Bossiaea obcordata
1/
98
Bossiaea obcordata
Bossiaea scortechinii
Bossiaea flexuosa
1/99
Bossiaea buxifolia
Bossiaea buxifolia
Bossiaea arenicola
1/99
0.96/-
Bossiaea brownii
Bossiaea rhombifolia
Bossiaea rupicola
1/99
1/
98
Bossiaea carinalis
Bossiaea carinalis
Bossiaea heterophylla
1/81
1/99
Bossiaea cinerea
Bossiaea cinerea
1/100
Bossiaea rosmarinifolia
Bossiaea kiamensis
1/91
Bossiaea cordigera
Bossiaea lenticularis
Bossiaea foliosa
1/94
Platylobium alternifolium
Platylobium obtusangulum
1/97
Platylobium formosum
Platylobium formosum
Platylobium triangulare
1/98
Bossiaea cucullata
Bossiaea cucullata
0.97/-
Bossiaea dentata

Figure S1b

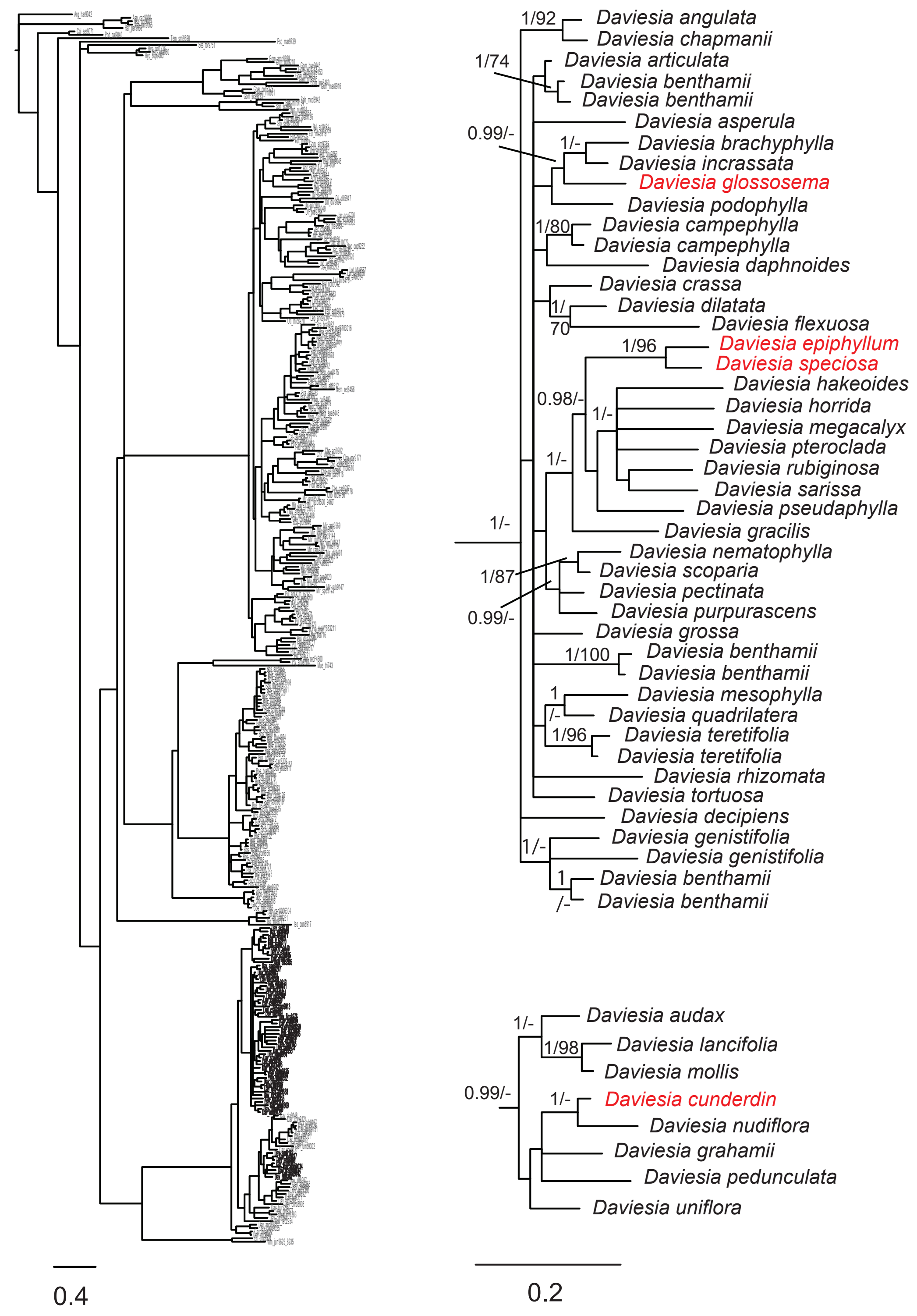

Daviesia angulata
Daviesia chapmanii
1/92
Daviesia articulata
1/74
Daviesia benthamii
Daviesia benthamii
Daviesia asperula
0.99/-
1/-
Daviesia brachyphylla
Daviesia incrassata
Daviesia glossosema
Daviesia podophylla
1/80
Daviesia campephylla
Daviesia campephylla
Daviesia daphnoides
Daviesia crassa
1/
70
Daviesia dilatata
Daviesia flexuosa
1/96
Daviesia epiphyllum
Daviesia speciosa
0.98/-
Daviesia hakeoides
1/-
Daviesia horrida
Daviesia megacalyx
Daviesia pteroclada
Daviesia rubiginosa
Daviesia sarissa
Daviesia pseudaphylla
1/-
Daviesia gracilis
1/-
Daviesia nematophylla
1/87
Daviesia scoparia
Daviesia pectinata
0.99/-
Daviesia purpurascens
Daviesia grossa
1/100
Daviesia benthamii
Daviesia benthamii
1
/-
Daviesia mesophylla
Daviesia quadrilatera
1/96
Daviesia teretifolia
Daviesia teretifolia
Daviesia rhizomata
Daviesia tortuosa
Daviesia decipiens
1/-
Daviesia genistifolia
Daviesia genistifolia
1
/-
Daviesia benthamii
Daviesia benthamii
1/-
Daviesia audax
1/98
Daviesia lancifolia
Daviesia mollis
0.99/-
1/-
Daviesia cunderdin
Daviesia nudiflora
Daviesia grahamii
Daviesia pedunculata
Daviesia uniflora
0.4
0.2

Figure S1c

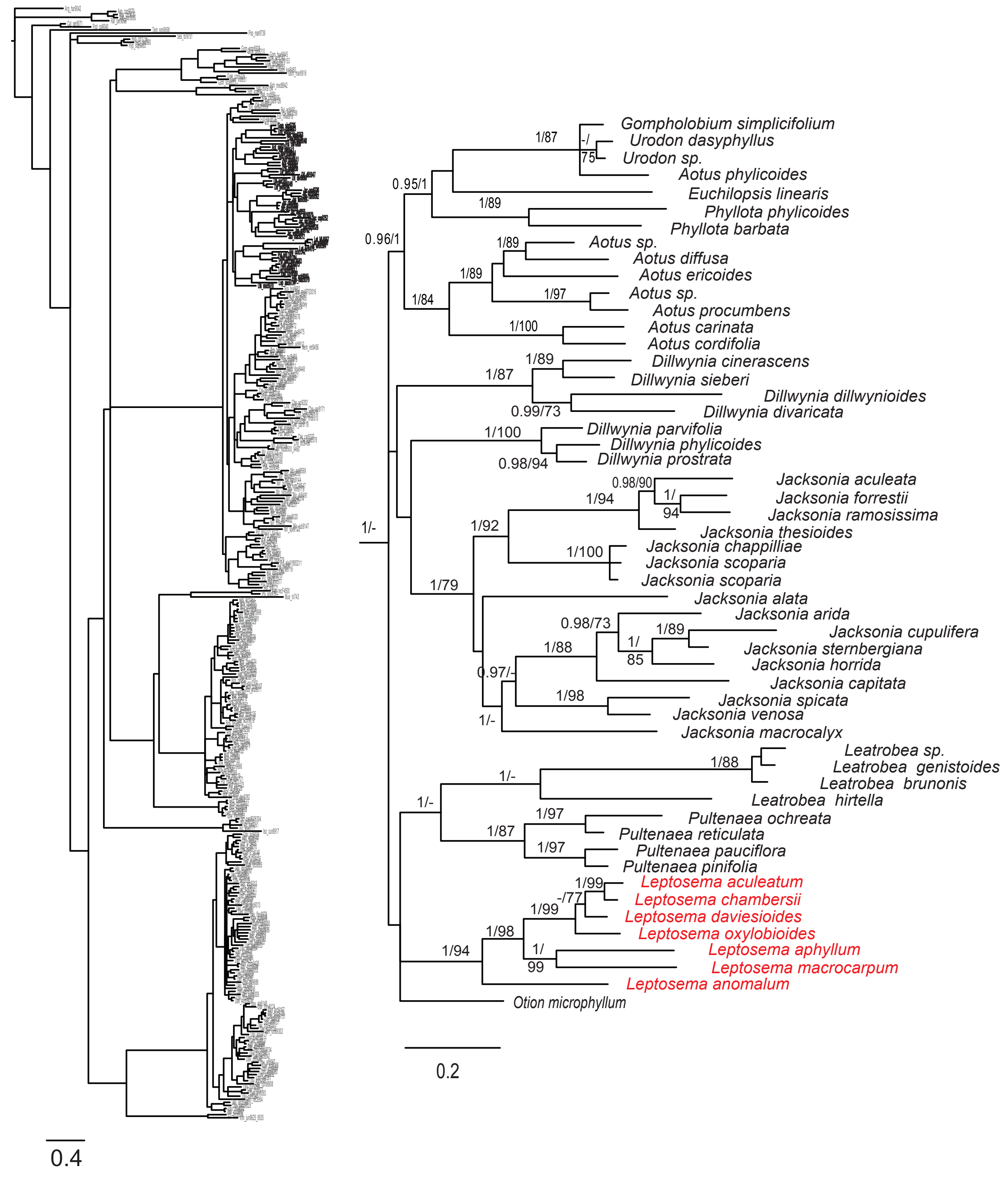

Gompholobium simplicifolium
Urodon dasyphyllus
Urodon sp.
Aotus phylicoides
Euchilopsis linearis
Phyllota phylicoides
Phyllota barbata
Aotus sp.
Aotus diffusa
Aotus ericoides
Aotus sp.
Aotus procumbens
Aotus carinata
Aotus cordifolia
Dillwynia cinerascens
Dillwynia sieberi
Dillwynia dillwynioides
Dillwynia divaricata
Dillwynia parvifolia
Dillwynia phylicoides
Dillwynia prostrata
Jacksonia aculeata
Jacksonia forrestii
Jacksonia ramosissima
Jacksonia thesioides
Jacksonia chappilliae
Jacksonia scoparia
Jacksonia scoparia
Jacksonia alata
Jacksonia arida
Jacksonia cupulifera
Jacksonia sternbergiana
Jacksonia horrida
Jacksonia capitata
Jacksonia spicata
Jacksonia venosa
Jacksonia macrocalyx
Leatrobea sp.
Leatrobea genistoides
Leatrobea brunonis
Leatrobea hirtella
Pultenaea ochreata
Pultenaea reticulata
Pultenaea pauciflora
Pultenaea pinifolia
Leptosema aculeatum
Leptosema chambersii
Leptosema daviesioides
Leptosema oxylobioides
Leptosema aphyllum
Leptosema macrocarpum
Leptosema anomalum
Otion microphyllum
1/87
-/
75
0.95/1
1/89
0.96/1
1/89
1/89
1/84
1/97
1/100
1/89
1/87
0.99/73
1/100
0.98/94
0.98/90
1/94
1/
94
1/92
1/-
1/100
1/79
0.98/73
1/89
1/
85
1/88
0.97/-
1/98
1/-
1/88
1/-
1/-
1/97
1/87
1/97
1/99
-/77
1/99
1/98
1/
99
1/94
0.2
0.4

Figure S1d
