# Supplementary material for: Evolutionary consequences of shifts to bird-pollination in the Australian pea-flowered legumes (Mirbelieae and Bossiaeeae)
Source: BMC Evol Biol. 2014 Mar 7;14:43. doi: 10.1186/1471-2148-14-43 (PMC4015313; doi:10.1186/1471-2148-14-43)
Supplement: Additional file 2: Figure S2 — Bayesian estimate of Mirbelieae and Bossiaeeae phylogeny using ITS sequence data showing clades that include both bird (red) and bee (black) pollinator syndromes. Full tree is shown on left with partial tree (bold in full tree) on right of a: Gastrolobium, b: Bossiaea and Platylobium, c: Daviesia and d: Jacksonia and Leptosema. Posterior probabilities (PP > 0.95) from Bayesian analysis and bootstrap support of bipartitions (BS > 0.70) from maximum likelihood analysis are shown on branches. Scale bar represents substitutions per site. [file 1471-2148-14-43-S2.pdf]

Additional file 2 Figure S2: Bayesian estimate of Mirbelieae and Bossiaeeae phylogeny using ITS sequence data showing clades that include both bird (red) and bee (black) pollinator syndromes. Full tree is shown on left with partial tree (bold in full tree) on right of a: *Gastrolobium*, b: *Bossiaea* and *Platylobium*, c: *Daviesia* and d: *Jacksonia* and *Leptosema*. Posterior probabilities (*PP* > 0.95) from Bayesian analysis and bootstrap support of bipartitions (BS > 0.70) from maximum likelihood analysis are shown on branches. Scale bar represents substitutions per site.

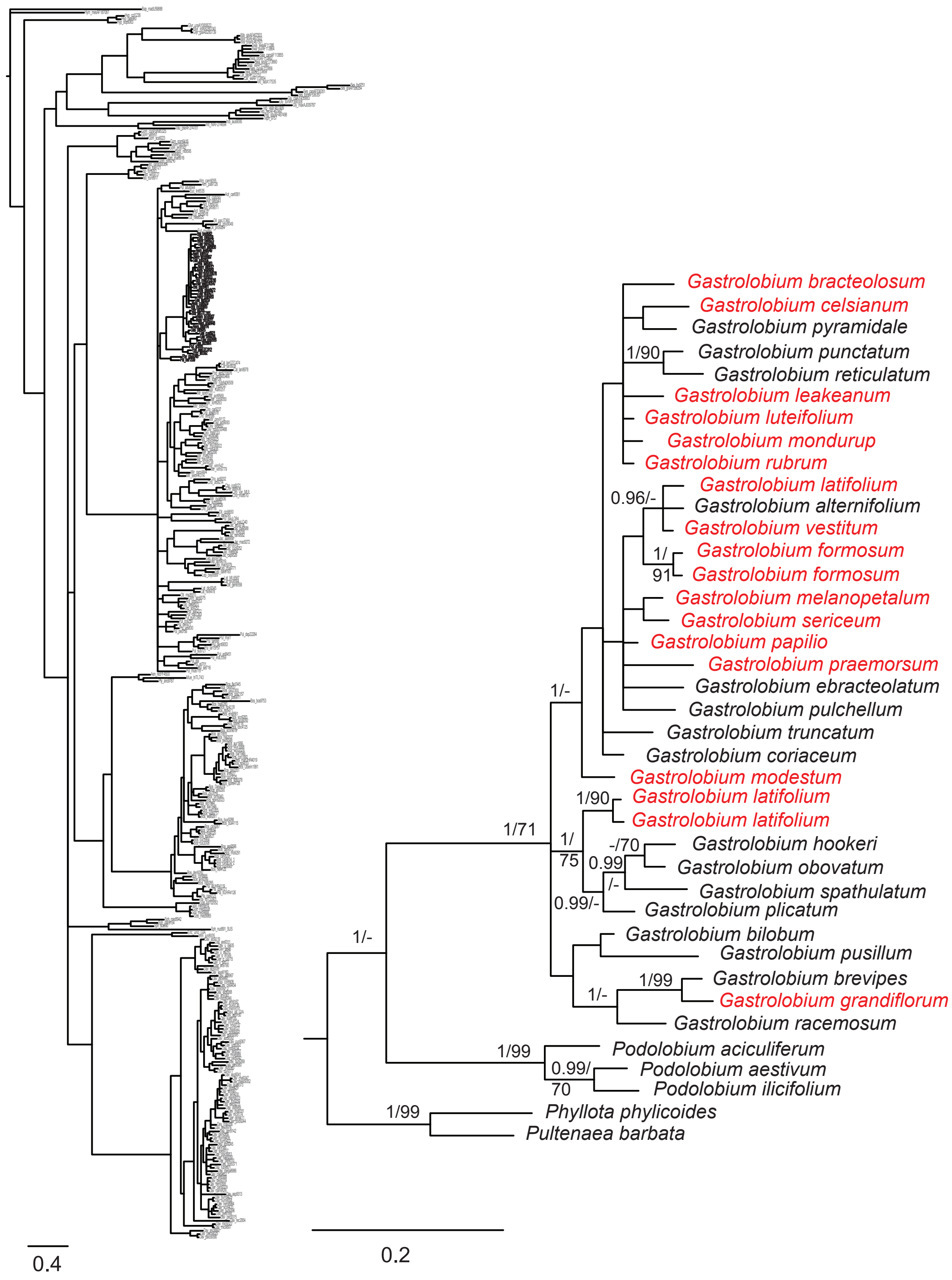
Gastrolobium bracteolosum
Gastrolobium celsianum
Gastrolobium pyramidale
1/90
Gastrolobium punctatum
Gastrolobium reticulatum
Gastrolobium leakeanum
Gastrolobium luteifolium
Gastrolobium mondurup
Gastrolobium rubrum
Gastrolobium latifolium
0.96/-
Gastrolobium alternifolium
Gastrolobium vestitum
1/
91
Gastrolobium formosum
Gastrolobium formosum
Gastrolobium melanopetalum
Gastrolobium sericeum
Gastrolobium papilio
Gastrolobium praemorsum
Gastrolobium ebracteolatum
Gastrolobium pulchellum
1/-
Gastrolobium truncatum
Gastrolobium coriaceum
Gastrolobium modestum
1/90
Gastrolobium latifolium
Gastrolobium latifolium
1/71
1/
75
-/70
Gastrolobium hookeri
0.99
Gastrolobium obovatum
/-
Gastrolobium spathulatum
0.99/-
Gastrolobium plicatum
1/-
Gastrolobium bilobum
Gastrolobium pusillum
1/99
Gastrolobium brevipes
Gastrolobium grandiflorum
1/-
Gastrolobium racemosum
1/99
Podolobium aciculiferum
0.99/
70
Podolobium aestivum
Podolobium ilicifolium
1/99
Phyllota phylicoides
Pultenaea barbata
0.4
0.2

Figure S2a

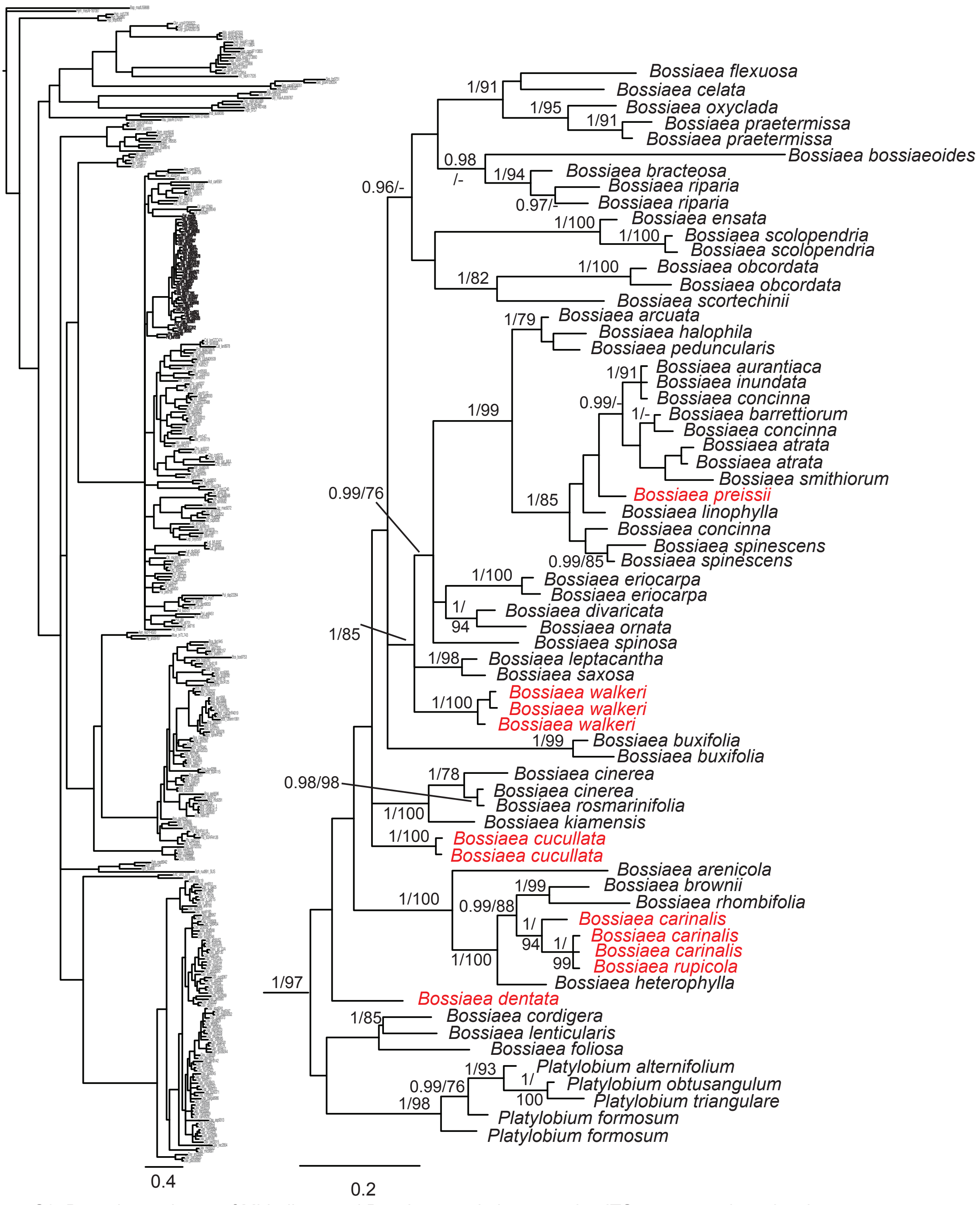
Bossiaea flexuosa
1/91
Bossiaea celata
1/95
Bossiaea oxyclada
1/91
Bossiaea praetermissa
Bossiaea praetermissa
0.98
/-
Bossiaea bossiaeoides
Bossiaea bracteosa
1/94
Bossiaea riparia
0.96/-
0.97/-
Bossiaea riparia
1/100
Bossiaea ensata
1/100
Bossiaea scolopendria
Bossiaea scolopendria
1/100
Bossiaea obcordata
1/82
Bossiaea obcordata
Bossiaea scortechinii
1/79
Bossiaea arcuata
Bossiaea halophila
Bossiaea peduncularis
Bossiaea aurantiaca
1/91
Bossiaea inundata
Bossiaea concinna
0.99/-
1/99
1/-
Bossiaea barrettiorum
Bossiaea concinna
Bossiaea atrata
Bossiaea atrata
Bossiaea smithiorum
0.99/76
Bossiaea preissii
1/85
Bossiaea linophylla
Bossiaea concinna
Bossiaea spinescens
0.99/85
Bossiaea spinescens
1/100
Bossiaea eriocarpa
Bossiaea eriocarpa
1/
94
Bossiaea divaricata
Bossiaea ornata
1/85
Bossiaea spinosa
1/98
Bossiaea leptacantha
Bossiaea saxosa
1/100
Bossiaea walkeri
Bossiaea walkeri
Bossiaea walkeri
1/99
Bossiaea buxifolia
Bossiaea buxifolia
1/78
Bossiaea cinerea
0.98/98
Bossiaea cinerea
Bossiaea rosmarinifolia
1/100
Bossiaea kiamensis
1/100
Bossiaea cucullata
Bossiaea cucullata
Bossiaea arenicola
1/99
Bossiaea brownii
1/100
Bossiaea rhombifolia
0.99/88
1/
Bossiaea carinalis
1/
94
Bossiaea carinalis
Bossiaea carinalis
99
1/100
Bossiaea rupicola
Bossiaea heterophylla
1/97
Bossiaea dentata
1/85
Bossiaea cordigera
Bossiaea lenticularis
Bossiaea foliosa
1/93
Platylobium alternifolium
1/
0.99/76
Platylobium obtusangulum
100
Platylobium triangulare
1/98
Platylobium formosum
Platylobium formosum
0.4
0.2

Figure S2b

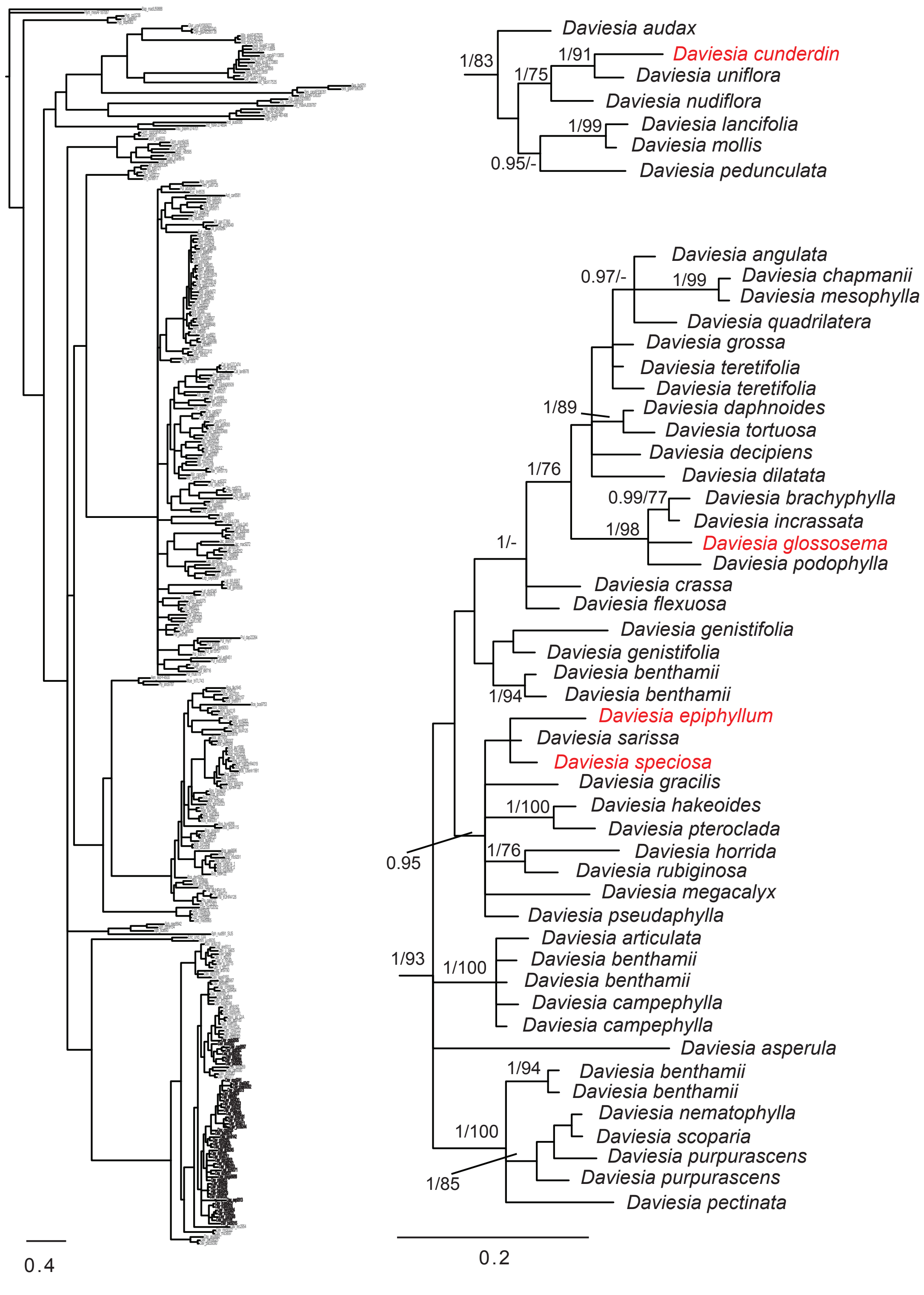
Daviesia audax
1/83
1/91
Daviesia cunderdin
Daviesia uniflora
1/75
Daviesia nudiflora
1/99
Daviesia lancifolia
Daviesia mollis
0.95/-
Daviesia pedunculata
Daviesia angulata
0.97/-
1/99
Daviesia chapmanii
Daviesia mesophylla
Daviesia quadrilatera
Daviesia grossa
Daviesia teretifolia
Daviesia teretifolia
1/89
Daviesia daphnoides
Daviesia tortuosa
Daviesia decipiens
1/76
Daviesia dilatata
0.99/77
Daviesia brachyphylla
Daviesia incrassata
1/98
Daviesia glossosema
1/-
Daviesia podophylla
Daviesia crassa
Daviesia flexuosa
Daviesia genistifolia
Daviesia genistifolia
Daviesia benthamii
1/94
Daviesia benthamii
Daviesia epiphyllum
Daviesia sarissa
Daviesia speciosa
Daviesia gracilis
1/100
Daviesia hakeoides
Daviesia pteroclada
0.95
1/76
Daviesia horrida
Daviesia rubiginosa
Daviesia megacalyx
Daviesia pseudaphylla
Daviesia articulata
1/93
1/100
Daviesia benthamii
Daviesia benthamii
Daviesia campephylla
Daviesia campephylla
Daviesia asperula
1/94
Daviesia benthamii
Daviesia benthamii
Daviesia nematophylla
1/100
Daviesia scoparia
Daviesia purpurascens
1/85
Daviesia purpurascens
Daviesia pectinata
0.4
0.2

Figure S2c

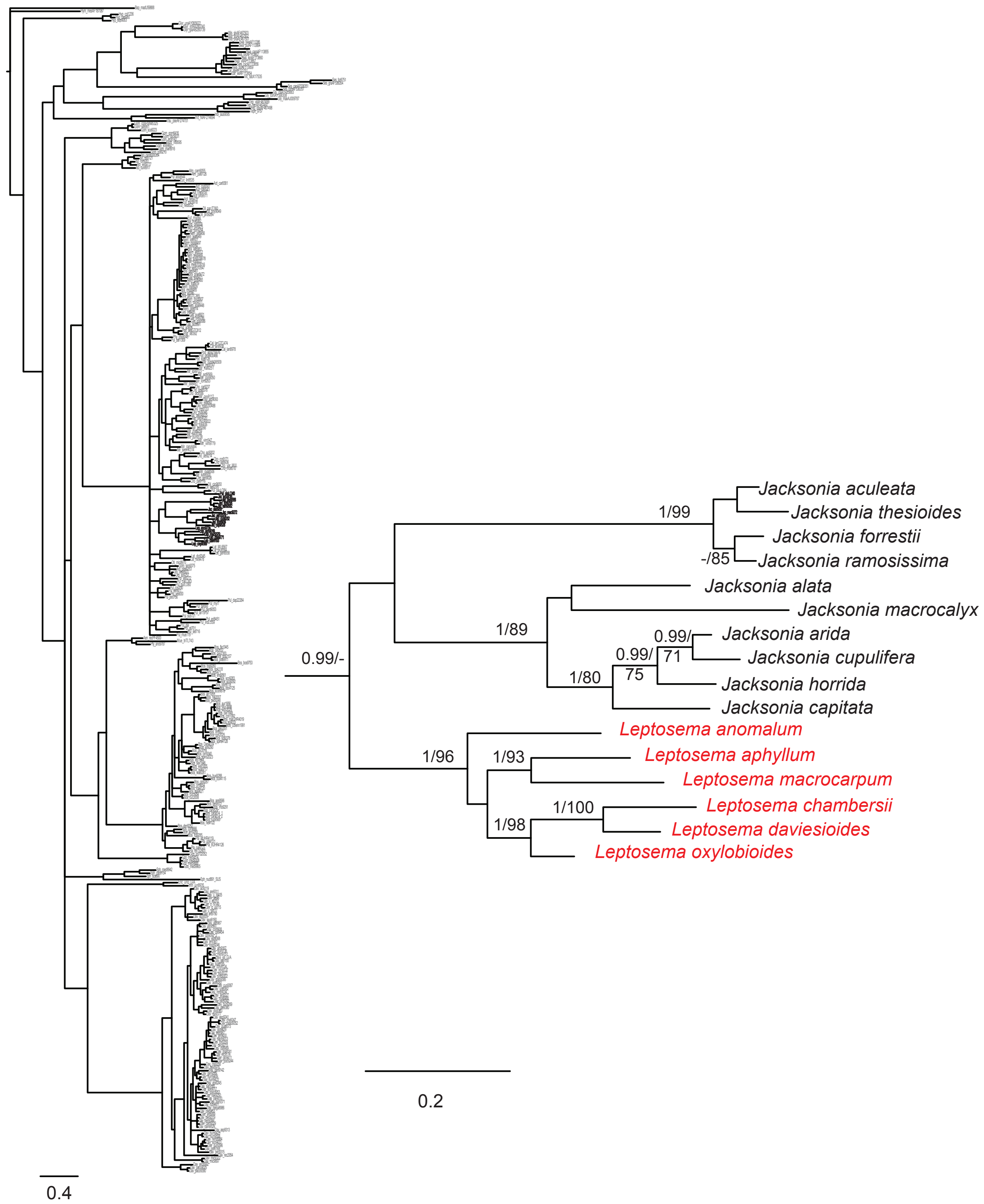

0.4
1/99
Jacksonia aculeata
Jacksonia thesioides
Jacksonia forrestii
-/85
Jacksonia ramosissima
Jacksonia alata
Jacksonia macrocalyx
1/89
0.99/
Jacksonia arida
0.99/
71
Jacksonia cupulifera
0.99/-
1/80
75
Jacksonia horrida
Jacksonia capitata
Leptosema anomalum
1/96
1/93
Leptosema aphyllum
Leptosema macrocarpum
1/100
Leptosema chambersii
1/98
Leptosema daviesioides
Leptosema oxylobioides
0.2

Figure S2d
